# Supplementary material for: Optimisation of Biomass Production and Nutritional Value of Two Marine Diatoms (Bacillariophyceae), Skeletonema costatum and Chaetoceros calcitrans
Source: Biology (Basel). 2022 Apr 14;11(4):594. doi: 10.3390/biology11040594 (PMC9024967; doi:10.3390/biology11040594)
Supplement: Supplementary file 1 [file biology-11-00594-s001.zip › biology-1629837-supplementary.pdf]

Table S1: Supplementary table to figure 1. *S. costatum* growth performance in terms of biomass dry weight (g L^{-1}) obtained in 1-L bubble column PBRs in order to optimize the supply of a specific nutrient (silicate, nitrate, phosphate, iron) or micronutrient concentrations (*n*=3). Values are represented as mean ± standard deviation.

**Silicate trial (g L^{-1} DW)**				
**Days**	0.4 mM	0.8 mM	1.2 mM	2.4 mM
**0**	0.16 ± 0.00	0.18 ± 0.00	0.18 ± 0.00	0.16 ± 0.00
**1**	0.44 ± 0.02	0.49 ± 0.02	0.47 ± 0.02	0.49 ± 0.01
**3**	0.86 ± 0.04	1.61 ± 0.11	1.72 ± 0.07	1.77 ± 0.01
**4**	1.24 ± 0.09	2.04 ± 0.06	2.08 ± 0.14	2.32 ± 0.09
**6**	1.86 ± 0.11	2.73 ± 0.13	2.55 ± 0.06	3.07 ± 0.13
**7**	2.02 ± 0.03	3.09 ± 0.22	3.04 ± 0.01	3.52 ± 0.17
**Nitrate trial (g L^{-1} DW)**				
**Days**	1 mM	2 mM	4 mM	8 mM
**0**	0.19 ± 0.00	0.18 ± 0.00	0.18 ± 0.00	0.18 ± 0.00
**1**	0.43 ± 0.01	0.42 ± 0.01	0.43 ± 0.01	0.42 ± 0.01
**3**	1.21 ± 0.03	1.65 ± 0.00	1.64 ± 0.08	1.71 ± 0.09
**4**	1.16 ± 0.06	1.63 ± 0.04	2.41 ± 0.11	2.54 ± 0.10
**7**	1.53 ± 0.09	2.20 ± 0.18	3.18 ± 0.07	3.38 ± 0.14
**Phosphate trial (g L^{-1} DW)**				
**Days**	50 μM	100 μM	200 μM	400 μM
**0**	0.18 ± 0.00	0.18 ± 0.00	0.18 ± 0.00	0.18 ± 0.00
**1**	0.47 ± 0.01	0.45 ± 0.01	0.45 ± 0.00	0.44 ± 0.03
**3**	1.48 ± 0.04	1.54 ± 0.12	1.35 ± 0.06	1.18 ± 0.05
**4**	2.89 ± 0.07	2.94 ± 0.03	2.65 ± 0.07	2.47 ± 0.11
**7**	3.45 ± 0.20	3.72 ± 0.07	3.38 ± 0.25	3.52 ± 0.10
**Iron trial (g L^{-1} DW)**				
**Days**	1 mM	2 mM	4 mM	8 mM
**0**	0.16 ± 0.00	0.18 ± 0.00	0.18 ± 0.00	0.18 ± 0.00
**1**	0.44 ± 0.01	0.44 ± 0.02	0.42 ± 0.01	0.43 ± 0.02
**3**	1.92 ± 0.01	1.89 ± 0.01	1.69 ± 0.15	1.69 ± 0.17
**4**	2.54 ± 0.09	2.59 ± 0.06	2.31 ± 0.12	2.45 ± 0.19
**7**	2.49 ± 0.12	2.56 ± 0.10	2.26 ± 0.20	2.40 ± 0.08
**Micronutrient's trial (g L^{-1} DW)**				
**Days**	0.5 mL L^{-1}	1 mL L^{-1}	2 mL L^{-1}	4 mL L^{-1}
**0**	0.19 ± 0.00	0.19 ± 0.00	0.19 ± 0.00	0.19 ± 0.00
**1**	0.50 ± 0.00	0.50 ± 0.01	0.50 ± 0.01	0.50 ± 0.00
**3**	1.88 ± 0.05	1.84 ± 0.03	1.68 ± 0.07	1.87 ± 0.00
**4**	2.54 ± 0.00	2.51 ± 0.02	2.34 ± 0.13	2.48 ± 0.02
**7**	3.09 ± 0.10	3.05 ± 0.05	2.86 ± 0.10	3.09 ± 0.07

Table S2: Supplementary table to figure 2. *C. calcitrans* growth performance in terms of biomass dry weight (g L^{-1}) obtained in 1-L bubble column PBRs in order to optimize the supply of a specific nutrient (silicate, nitrate, phosphate, iron) or micronutrient concentrations (*n*=3). Values are represented as mean ± standard deviation.

**Silicate trial (g L^{-1} DW)**				
**Days**	0.4 mM	0.8 mM	1.2 mM	2.4 mM
**0**	0.12 ± 0.00	0.12 ± 0.00	0.12 ± 0.00	0.12 ± 0.00
**1**	0.39 ± 0.01	0.41 ± 0.00	0.41 ± 0.01	0.42 ± 0.01
**3**	0.67 ± 0.02	0.89 ± 0.03	0.95 ± 0.03	1.00 ± 0.03
**5**	0.68 ± 0.01	1.56 ± 0.02	1.64 ± 0.01	1.77 ± 0.05
**7**	0.76 ± 0.03	1.61 ± 0.03	2.03 ± 0.06	2.05 ± 0.08
**Nitrate trial (g L^{-1} DW)**				
**Days**	1 mM	2 mM	4 mM	8 mM
**0**	0.12 ± 0.00	0.12 ± 0.00	0.12 ± 0.00	0.12 ± 0.00
**1**	0.29 ± 0.00	0.30 ± 0.00	0.29 ± 0.00	0.29 ± 0.00
**3**	0.46 ± 0.01	0.97 ± 0.01	0.95 ± 0.00	0.92 ± 0.07
**5**	0.55 ± 0.05	1.20 ± 0.05	1.60 ± 0.04	1.53 ± 0.07
**7**	0.73 ± 0.03	1.13 ± 0.02	2.17 ± 0.02	2.03 ± 0.02
**Phosphate trial (g L^{-1} DW)**				
**Days**	50 μM	100 μM	200 μM	400 μM
**0**	0.04 ± 0.00	0.04 ± 0.00	0.04 ± 0.00	0.04 ± 0.00
**1**	0.30 ± 0.00	0.30 ± 0.01	0.29 ± 0.00	0.25 ± 0.00
**3**	1.12 ± 0.04	1.20 ± 0.05	1.08 ± 0.06	1.01 ± 0.09
**5**	1.53 ± 0.02	1.68 ± 0.01	1.62 ± 0.05	1.53 ± 0.09
**7**	2.32 ± 0.05	2.44 ± 0.02	2.31 ± 0.06	2.19 ± 0.08
**Iron trial (g L^{-1} DW)**				
**Days**	10 μM	20 μM	40 μM	80 μM
**0**	0.11 ± 0.00	0.12 ± 0.00	0.12 ± 0.00	0.12 ± 0.00
**1**	0.29 ± 0.00	0.30 ± 0.00	0.30 ± 0.00	0.31 ± 0.00
**3**	1.05 ± 0.01	1.04 ± 0.03	1.01 ± 0.02	1.03 ± 0.01
**5**	1.39 ± 0.02	1.45 ± 0.02	1.62 ± 0.02	1.61 ± 0.03
**7**	1.66 ± 0.06	1.76 ± 0.02	1.93 ± 0.06	2.09 ± 0.02
**Micronutrients trial (g L^{-1} DW)**				
**Days**	0.5 mL L^{-1}	1 mL L^{-1}	2 mL L^{-1}	4 mL L^{-1}
**0**	0.12 ± 0.00	0.12 ± 0.00	0.12 ± 0.00	0.12 ± 0.00
**1**	0.30 ± 0.00	0.36 ± 0.00	0.36 ± 0.00	0.36 ± 0.00
**3**	1.19 ± 0.07	1.14 ± 0.03	1.32 ± 0.03	1.22 ± 0.04
**5**	1.78 ± 0.01	1.75 ± 0.05	1.80 ± 0.05	1.75 ± 0.06
**7**	1.99 ± 0.05	1.96 ± 0.00	1.95 ± 0.06	1.92 ± 0.16
